# From kinship networks to culture of relatedness: a shift of safety nets during health pandemics in the kenyan context

**DOI:** 10.3389/fpubh.2023.1062962

**Published:** 2023-06-19

**Authors:** Agnetta Adiedo Nyabundi

**Affiliations:** The Center for the Advancement of Scholarship, University of Pretoria, Pretoria, South Africa

**Keywords:** kinship, community safety nets, coping mechanisms, pandemics, COVID-19

## Abstract

Evidence suggests that, during pandemics such as COVID-19, people with low incomes within developing countries suffered disproportionately. Households across countries differentially experienced the socio-economic impact of the pandemic. In sub-Saharan Africa, the extended family and the community have provided valuable support in crises, given that state-administered backing may not be sufficient or may differ from the family's expectations. Many studies have been conducted on community safety nets, yet little description and understanding of community safety nets has been provided. The components of the non-formal safety nets are yet to be adequately defined or evaluated for effectiveness. Traditional family and community safety nets have been under stress due to the impact of COVID-19. Many countries, including Kenya, have associated COVID-19 with an increased number of households facing social and economic crises. Families and communities got fatigued due to the extended period and the further strain the pandemic had on individuals and societies. Utilizing existing literature on the socio-economic impact of COVID-19 in Kenya and the roles and perceptions of community safety nets, this paper seeks to explain the roles and perceptions of social relationships and kinship networks as safety nets in Africa, specifically in the Kenyan context. This paper employs the concept of culture of relatedness to understand the informal safety nets in Kenya better. During the COVID-19 pandemic, individuals strengthened the previously weakened kinship structures. They addressed some of the challenges experienced within the networks through the involvement of neighbors and friends embracing the culture of relatedness. Therefore, government strategies for social support during pandemics need to design programs to strengthen the community safety nets that remained resilient throughout the health crisis.

## Introduction

A policy brief by the Strategic Policy Advisory Unit (Unit, S.P.A) noted that the COVID-19 pandemic had had direct and indirect effects on the socio-economic levels of households. This policy brief further states that the impact of COVID-19 varies from an income earner in the family falling ill, which leads to a drop in the ratio of active members to dependents to when it is the dependents of the income earner who fall sick (1). The effects of COVID-19 may be intensified by lost avenues for earning a source of livelihood and taking care of the ailing family member or even by funeral expenses incurred upon death (1). A policy brief noted that ill-health and limited resilience capacities could create multiplier effects (1). The COVID-19 pandemic is a more significant health crisis since its impact has been felt at the core of societies and economies (2). Despite the variations in its effects on countries, COVID-19 will likely increase poverty and inequalities globally, bringing a greater urgency toward achieving Sustainable Development Goals (S.D.G.s).

The Organization for Economic Co-operation and Development (OECD) noted that countries across the globe introduced stringent confinement measures during the pandemic (3). The core aim of the confinement measures was to reduce and contain the spread of the COVID-19 virus. This act of individuals' confinement was also geared toward reducing the unbearable pressure on hospitals and, ultimately, reducing the pandemic's death toll (3). Accompanying the confinement measures, as noted by OECD, were side effects such as a significant supply shock, as workers were forced to stay home and many businesses were temporarily shut down. Another side effect was the reduced demand for many goods and services as households and companies could no longer physically or financially afford them. In this unprecedented situation, countries grappled with minimizing the lockdown's impact on their citizens' livelihoods. Governments discussed how to support the citizens, with all debates anchored on sustainability (1). The fiscal sustainability worries of Governments were put on hold as policymakers geared toward averting more profound socio-economic crises.

In the Kenyan context, the speed and severity of the pandemic shock have been met with unprecedented levels of support, both in-depth and scope (1). Like other health pandemics, COVID-19 increased the short-term shocks and long-duration stresses in Kenya as in other developing countries (4). As Arnall et al. (4) noted, these shocks and stresses result from economic decline, increased poverty, and deteriorating living conditions. The household responses to the pandemic have depended on the household's available assets, the economic context, past migration history, and contemporary rural links. The solutions to the shocks and stresses of COVID-19 have been influenced by disease prevalence and its effect on the household. The kind of support received also depends on the social/ethnic group a family belongs to, with associated kinship patterns (4). Marriage and associations individuals hold within their households and beyond also determine the kind of support individuals receive as a response to the stresses and strains of life resulting from COVID-19. The answers to the shocks and anxieties due to the pandemic depended on the government's capacity to deliver services and activities to non-governmental organizations. Despite all the structures put in place to address the socio-economic stresses resulting from COVID-19, the sustainability aspect of this support still needs to be evaluated.

During the COVID-19 pandemic, the Government of Kenya helped support families through specific initiatives geared toward alleviating or reducing the shocks and stresses of the pandemic (1). The cash value of the grants was, however, relatively small to address the significant impact of the pandemic on households (1). It is important to note further that the social protection offered by the Government of Kenya had previously not been set apart for addressing shocks and stresses that resulted from COVID-19. The Kenyan Government's social protection manages socio-economic needs such as hunger, the old, orphans, and vulnerable communities (1). Therefore, it puts pressure on the kinship networks, both those based on genealogy and eventually involving those not related by genealogy, including friends, neighbors, and community members working together to assist those in need.

Researchers noted that informal community arrangements generally work well under certain circumstances (4). They can, however, begin to break down due to stress and strain due to prolonged or widespread seasons of crisis, as the one experienced due to the long duration of COVID-19 (4). Moser (5) shows how the pressures of economic crisis can exert opposing forces on local transfer relationships, strengthening them through increasing reciprocity networks, and eroding them, as households' ability to cope deteriorates and community trust breaks down. According to Reece (6), families must find ways to reconfigure their relationship, thus incorporating their growth and reproduction through sufficient distance within the kinship structure. During pandemics and times of strain and stress, the question is how families will create space, yet it is a time of need. Given the long duration that COVID-19 has been with us, it is essential to look at the impact of the pandemic stresses on kinship safety nets. The COVID-19 period was when family members needed each other most. Yet, it was a period of immense socio-economic stress and care burden in Kenya—understanding the perception of individuals on the structure and function of kinship networks in the context of COVID-19 in Kenya. This paper, therefore, sets out to utilize existing literature and theories on kinship and safety nets to identify and describe the components of community safety nets and how they have changed over time. It also endeavors to further understand the roles and perceptions of social relationships and kinship networks as safety nets within Africa, specifically focusing on Kenya.

## Toward understanding community safety nets during a pandemic

Researchers challenged the safety net discourse with the emergence of social protection in the late 1980s and early 1990s (7). Devereux and Sabates-Wheeler (7) further documented that during the 1990s, thinking on livelihoods, risk, vulnerability, and the multidimensional nature of poverty became more pronounced. Studies increasingly criticized safety nets as residualist and paternalistic, proposing more sophisticated alternatives (7). In low-income countries, social protection continues to be perceived by governments and donors as providing unsustainable transfers to individuals unwilling to work and transform their socio-economic status. Social protection has further been deemed a diversion of scarce public resources from productive investment, which should be used for economic growth (7). On the contrary, it is essential to note that when individuals are empowered and enabled, they can contribute to the economic growth of their societies. The effects of diseases and sicknesses such as COVID-19 can be a great source of strain, impeding individual productivity and contribution toward economic development.

Development agencies have, however, continued to conceptualize social protection mainly as a public response to livelihood shocks (7). This article views social protection with a broader lens away from the perception of resource transfer. It encompasses the dimension of social services provision toward reducing vulnerability and risk of individuals due to the impact of disease and pandemic. According to an International Labor Organization (I.L.O.) report^1^ on social protection, state-administered social insurance within sub-Saharan Africa is insufficient. I.L.O. ^1^ further notes that one of the most pressing challenges for social protection in Africa is access to health care, mainly due to financial constraints. In Kenya and Senegal, the government pays 45 percent of total health expenditure as out-of-pocket payments ^1^. The report further mentions that catastrophic health expenditure is one of the significant poverty risks for individuals and their families. Paying for medicine and health care may force families into poverty for years. These challenges prompt further support through kinship ties to enable household members to access economic, social, psychological, and emotional support from relatives, friends, and neighbors in times of need. According to Reece (6), Societies expect families in many contexts to persist indefinitely while accommodating massive socio-political change and great upheavals such as pandemics.

The informal safety nets, including family members, neighbors, friends, and community associations, contribute to household support systems, especially during pandemics such as COVID-19. However, these community safety nets are inadequately described and poorly understood, as noted by Foster (8). The key pillars of the informal social security provision or community safety nets include reciprocity and social cohesion (7). A study conducted in Uganda noted that to guarantee sufficient social protection in good and bad times to all members of any ethnic nationality, the acts of reciprocity, altruism, social cohesion, and personal intimacies were inevitable in ensuring equity and social justice (9). Researchers have nevertheless criticized the view based on often engendering relations of subservience and dependence (10). Mkhwanazi and Manderson (11), in their book “Connected Lives,” a study conducted in South Africa, have, however, noted that kinship and residence, families, and households connect and give meaning to lives. They further state that families and households care for basic human needs: food and shelter, reproduction, and social and daily production. The care provided to individuals occurs whether the family is biologically based or chosen, heterosexual or otherwise, large or small, matrilineal or patrilineal, nuclear or extended.

Households might draw their core members from marriage and blood ties or intentionally have members drawn together through love and affective ties. Families can be very small and stable or extremely large and fluid, spreading and shrinking as personal circumstances and domestic and local economics allow (11). They (11) also underscore the importance of families in providing practical and emotional ties for people to feel supported; households give the settings in which these ties are lived out daily. Beyond and within households, families provide the structures and resources for everyday life and the context through which people manage intermittent, often minor but sometimes catastrophic, health, economic, and other crises (12). According to Mkhwanazi and Manderson (11), families are at the heart of birth, death, health, and illness. They (11) further find out that it is within families and households that biology and sociality are intertwined.

Despite the feelings of subserviency and dependence, as shown by Davies (10) in a critique of the acts of reciprocity and altruism that individuals receive during a pandemic and the socio-economic stress accompanying a pandemic, families, households and communities have proven to remain intact to support each other. Kin and family, in this sense, are idealized as sources of intimacy and belonging (6). Reece (6), in the study on pandemic kinship: Families, intervention and social change in Botswana's time of AIDS, notes that the idealized intimacy brings unique risks and danger or influx in the sense of belonging. Therefore, despite the solidarity and deep interdependency within the kinship, tensions emerge given the diverse modes of personhood (6). Given the extended period of the pandemic, there is a need to establish the role and perceptions of kinship support. Its effect on the Kenyan context was such that most of the kin relied on their savings, incomes, and help from friends to meet COVID-19-related expenses such as hospital bills. During the early stages of the pandemic, most health insurance companies did not cover COVID-19 treatment and, worse off, funeral costs in the cases of death of kin within the Kenyan context, impacting heavily on people's lives.

## An analysis of the role and perceptions of social relationships and kinship networks as safety nets

Studies have shown that social relationships are linked to better health in several ways; however, this is only in theory and may vary in practice (13). Heady and Grandits (13) further mention that social relationships play several vital roles, including providing emotional benefits such as intimacy, a sense of belonging, and self-esteem. Through physical assistance, such as money, goods, services, and advice, social relationships continue to offer instrumental help (14). Durkheim's (15) studies on the association between social isolation and suicide included reports on the benefits of social networks to health care, for example, how social support and social engagement reduce mortality risks and disability (16–19), improve disease recovery rates (20), and promote cognitive development and function (21, 22). The anthropology of kinship has majorly focused on aspects of solidarity and deep interdependency, as noted by Reece (6). Reece (6) further states that tensions emerge within kinship, given the diverse modes of personhood. Although most studies focus on the beneficial effects of social relationships, networks may also contain relationships that negatively affect mental and physical health (23, 24). This further calls for analyzing social networks within their sociocultural context and their Influence on health and wellbeing.

Barnett (25) defines kinship networks as extended family, including biological relationships, genealogy, marriage, and other self-ascribed associations beyond the nuclear family. Barnett (25) further notes the conceptualization of kinship as socially and culturally constructed and a maintained network of individuals in constant flux and not fixed on the genealogical relationship. Therefore, biology, sexuality, and descendancy are no longer the sole defining factors in understanding kinship (25). There have been increasingly blurred boundaries between kinship, community, and friendship networks. Historically, marriage and kinship are the most significant factors that organize and structure people's economic, political, and social lives (25). Marriage, however, was not for the benefit of the husband and wife only, but it played a social function with secondary consideration to women's and children's needs (25). Barnett (25) further notes that marriage and the consequently emerging kinship ties and networks would help raise capital, maintain privilege and family lines across generations, organize the division of labor, create political alliances, and define parent-children authority relationships.

Nevertheless, the family's role has changed over time and space. Changing marriage, cohabitation, divorce patterns, declining fertility, and aging populations affect the family's social security role. Barnett (25) notes that the emerging features of contemporary families are not particularly new. He (25) further asserts that numerous historical records of non-traditional family patterns existed, including; high divorce rates, extramarital sex, out-of-wedlock births, step-families, and rare occasions of culturally accepted same-sex marriages. Studies have mentioned families as the most important social support structures for all human beings worldwide (11). However, the dynamism within the form and role of kinship networks makes it necessary to establish the current range of strategies the kin explores during pandemics such as COVID-19.

Structural analysis of kinship networks maps the relationship between individuals. It examines social ties and the frequency of contacts, directness of interaction, network density, household composition, and generational exchanges, among other variables. Functional analysis of kinship networks focuses on the construction and maintenance of social ties; questions of reciprocity; and the kind and amount of support given and received by members of the network, including instrumental (care work, household help, and financial and material assistance) and expressive (socioemotional and psychological) support (25). Situating kinship and social support network studies within cultural contexts are essential to refine and extend the concept's understanding (26). This helps to refine and extend understanding of the concept (26). Kinship networks usually do not operate on market principles of exchange (most commonly money). Kin status instead comes with clear and well-defined rules of behavior and responsibility, albeit reciprocity holds kinship networks together. Reciprocity refers to members' ability to give back (25). Barnet (25) further notes that reciprocity is often approached from a utilitarian social exchange perspective, providing a challenging and complicated task of assessing value in networks based on affectionate ties and emotional attachment. For this reason, some researchers theorize reciprocity as a norm that brings on culturally determined obligations and governs desirable human relationship patterns (25). Anchors and network members actively construct perceptions and vocabularies of value outside the monetary realm and navigate a structurally determined landscape, evaluating and measuring each other's commitments, needs, intentions, and abilities compared to their own. Mann and Delap (27) noted that in Kenya, family and close friends cared for a regular part of childhood. Further to this study, it would be interesting to know the perceptions of the Kenyan community on the aspects of kinship support networks in the wake of COVID-19.

The Kenyan community is majorly patriarchal. In the patriarchal societies in Kenya, payment of bridewealth provided space and suitable resources for children and their mothers within the kin group (28). The husband was responsible for his wife's conjugal rights, while the entire community was responsible for socializing their children (29). In addition to the socialization process, the community was also responsible for supporting the orphaned children and widows (30). This communal kin assistance has, however, shifted due to socio-economic challenges and changes in the kinship structure and uncertainties during pandemics such as HIV/AIDS over time, forcing widows to seek alternative sources of support (30).

HIV/AIDS as a pandemic prompted the need to review kinship structures and roles during that time. During the COVID-19 pandemic, individuals in Kenya suffered economically, socially, and even psychologically (1). The disease burden and its impact on the few available resources significantly affected the family. For instance, at some point, most COVID-19 patients would not get hospital admission but had to be taken care of from home, yet some of the patients were the breadwinners of their families. Families had to take care of their kin independently within the home setting, which had also been suffering the COVID-19 socio-economic effects. This scenario also concurs with a study conducted in South Africa by Mkhwanazi and Manderson (11), who revealed that affective, social ties have continued to bring meaning to people's lives. They (11) noted that the power of family relations is irreplaceable despite any form of outsourced services, such as when caregivers, for example, nurses, come in to care for the sick. Hence the need to further look at the challenges the kinship networks have faced despite being the major resort of care for the sick and economic support.

The policy brief by the Strategic Policy Advisory Unit (Unit, S.P.A) (1) indicates that family networks, especially during the COVID-19 pandemic, were most often anchored and constructed by women. This brief (1) further noted that women faced the burden of care for extended family members and children when they were not in school during the COVID-19 pandemic. Nonetheless, even in female-anchored networks, men play important roles through instrumental and expressive support. About the Ebola disease, Mulvihill (31) noted that women were responsible for taking care of ill family members, exposing them to a higher risk of contracting the disease other than sacrificing their time too. The confinement measures that Governments introduced to help reduce the spread of COVID-19 entailed more people staying at home, burdening women with more household chores. Kenyan women, for example, account for 50.5% of the population (32) and spend 11.1 h on care work compared to only 2.9 h by men (33).

Oxfam (33) further states that at the pandemic's peak, Kenya's public and private health facilities faced the challenge of accommodating more patients due to the low capacity of the isolation wards. This challenge forced individuals to manage the infected persons at home, a caring process mainly by women. Women tend to be caregivers for the sick in healthcare settings and at home, which can expose them to more infectious agents than men (33). According to Mkhwanazi and Manderson (11), gender norms continue to dictate women's and men's roles. Furthermore, they (11) concur with the previous reports that society often sees women as the caretakers of the sick and domestic chores. At the same time, society deems to be responsible for income generation. Mkhwanazi and Manderson (11) further mention that there are instances when men take on caregiving when family support systems are thin, as has been the case during the COVID-19 pandemic.

## The current context of kinship networks in kenya: a shift to cultures of relatedness

The patriarchal society in Kenya has its kinship mainly based on blood ties. Traditionally, the Kenyan family is tasked with the provision of moral, ethical, spiritual, and cultural content to individuals (34). The family also meets the physical and emotional needs of its members. However, the family structure has experienced some changes due to strains in social relations. For example, during the H.I.V./AIDs pandemic in Kenya, orphaned and vulnerable children were fostered traditionally through kinship care (35). However, family and community support dwindled due to changes in population structures where economically productive populations drastically reduced due to H.I.V./AIDs (36). Fostering of kin is one of Africa's essential practices, including in Kenya. It entails the circulation of children to extended kin networks and communities (34). The kinship structure has often been regarded as a critical agent of care and protection for children (36, 37). However, this structure has weakened, forcing families and kin to opt for institutional care for their family members in crisis, including orphaned and vulnerable children (34).

The concept of the cultures of relatedness (2000) proposed by Janet Carsten examines relatedness as a broader concept of kinship, enlarging the analytical territory. It has opened the door to a general social contextualization of kinship (37). The concept of relatedness brings people to a new consciousness of their connections to others in a comparative context (37). Some of these connections may be valuable socially, materially, or affectively. Carsten (37) further notes that relationships may not always be decided on genealogy but can also be described in other ways. For example, among the Nuer people of Southern Sudan, there have been connections and disconnections of relatedness due to the profound social and political upheavals that they have faced through time. The Nuer relatedness has come to be understood by researchers through blood and cattle and the media of money, guns, and paper (37). It is necessary to understand how the phrases of relatedness are considered other than having the classic understanding of kinship (37). Studies have viewed the concept of relatedness as a dynamic process involving more than biological relations but more of the daily acts of taking care of each other, even in times of crisis, provoking a re-examination of what constitutes relations.

In the book Chapter Choosing kin: Sharing and subsistence in a Greenlandic hunting community, Nuttal argues that kinship is the foundation of social relatedness and social organization (38). Nuttal, in the book chapter, further notes that kinship is the fundamental organizing principle for subsistence activities in Kangersuasiaqa, a village in North West Greenland (38). Kinship is flexible and can be created by individuals and deactivated when individuals deem certain relationships unsatisfactory (39). During the socio-economic strain due to the COVID-19 pandemic, individuals forged relationships outside the kin to further strengthen the kin relationships by bringing stability in crises. All these acts that further the concept of kinship into cultures of relatedness need to be understood within specific ethnographic contexts. The idea of cultures of relatedness helps to view social support offered to the individuals not just by the kinship members from a genealogical perspective but also from other perspectives, such as support provided by friends and neighbors. Reece (6) notes that crises create, recalibrate, and produce kin relations. Howell (40) examines how individuals may address infertility problems through new reproductive technology (N.R.T.) or adoption. The process of adoption, as noted by Howell (40), assigns nakedness both literally and socially to the child. Howell (40) uses the term of the adopted child having been “de-kinned”-removed from “kinned” sociality, but eventually, through the new family, the children are “kinned.” This further shows how social relations are forged further and not only in times of crisis.

## Summary of findings

The kinship networks have always been helpful during pandemics such as COVID-19. The confinement measures and the hard-economic times resulting from the loss of income for most of the population meant that the family had to come in and support their kin. Though not well-described, families and kin structures could care for the sick at home when their fellow kin could not afford hospital care. Kinship structures supported family members to sustain their livelihoods by contributing to buying necessities for each other. Kin relations, neighborhood, and community structures were further strengthened and provided support and care for its members despite the negative impact of the pandemic. However, the long duration of the COVID-19 pandemic caused emotional, social, physical, and financial fatigue in families forcing individuals to forge new relations outside the family and the kin structure. Individuals reach out to friends and neighbors who are not related to them by blood but through social relations to get social support, given the insufficiency of the government's social support. Therefore, the shift occurred from kinship networks to cultures of relatedness and eventually social relatedness toward supporting each other during the COVID-19 pandemic.

## Conclusion

As a result of COVID-19, its long duration had significant social and economic stresses and strain in the Kenyan context. The Government alone could, however, not addresses these challenges. The kinship networks comprising family and close family friends helped to relieve individuals of the socio-economic challenges caused by COVID-19. There is a saying in Kenya that kinship relationships due to marriage are always complimented through friendship. There is a need to contextualize the various social support networks provided by family and kin relationships. During the pandemic, the support networks extended to neighbors and all social networks created by individuals apart from their families. The aspect of context is vital, given the dynamism experienced within the family and kinship system today. The changes in family and kin relations structure, coupled with the long duration of the pandemic, affected the levels of support. Therefore, further studies can be conducted within different contexts, especially where there have been drastic changes in the family structure and how these changes have affected social support during a pandemic.

## Limitations of the study

This paper can indeed help advise policy on social protection systems in Kenya. However, it is limited in its methodological approach, given that the current study did not engage in fieldwork to describe the kinship structure, its roles, and kin perception. However, given the socio-economic challenges families experienced during the pandemic, it was necessary to note that the kin structures were strengthened and further expanded outside the biological kin.

## Recommendations

1. It would be interesting for researchers to conduct a field study exploring the change of structures within the kinship networks in the wake of COVID-19 within the Kenyan context, given that kinship is socio-culturally constructed and maintained rather than based on genealogy.
2. There is a need to examine the structural dynamics within kinship networks significantly and how they have impacted the provision of safety nets among the different members of society.
3. The disruption and weakening of the non-formal intergenerational transfers have had a different picture during the COVID-19 pandemic. Kin relations, neighborliness, and community structures were strengthened further during COVID-19. This was evident through providing support and care for family members independently despite the pandemic's negative impact on individuals' lives. Therefore, in designing social protection programs to mitigate the socio-economic consequences of pandemics such as COVID-19, policymakers need to create programs that consider building socio-economic resources of households and strengthening community safety nets.
4. During a crisis, Governments help individuals through monetary transfers, material support, and services other community members offer (41). These kinds of assistance are not viable during pandemics such as the COVID-19 pandemic. Kin relations have therefore continued to help families recover and become self-sufficient in crises. The Government of Kenya needs to acknowledge and integrate the various roles played by the kinship networks as they provide valuable support even to the extent that the Government may not reach toward ensuring healthy lives and promoting wellbeing for all at every age.

## Author contributions

AN conceptualized and designed the final manuscript. This paper is part of the author's work at the African Research Universities Alliance conference in 2021.

## Footnotes

^1^ https://www.ilo.org/africa/areas-of-work/social-protection/lang--en/index.htm

## References

[1] Unit SPA. Articulating the Pathways of the Socio-Economic Impact of the Coronavirus (Covid-19) Pandemic on the Kenyan Economy 1, Special Policy Advisory Unit, UNDP, Kenya (2020).

[2] United Nations Economic Commission for Africa (UNECA). Economic Effects of the Covid-19 on Africa. Addis Ababa: UNECA (2020).

[3] OECD. Supporting People and Companies to Deal with the Covid-19 Virus: Options for an Immediate Employment and Social-Policy Response. (2020). Available online at www.oecd.org/employment/ (accessed November 23, 2022).

[4] Arnall A Furtado J Ghazoul J De Swardt C. Perceptions of informal safety nets: a case study from a South African informal settlement. Dev. Southern Afr. (2004) 21:443–60. 10.1080/0376835042000265432

[5] Moser CO. The asset vulnerability framework: reassessing urban poverty reduction strategies. World Dev. (1998) 26:1–19. 10.1016/S0305-750X(97)10015-8

[6] Reece KM. Pandemic Kinship (Vol. 67). Cambridge University Press (2022). 10.1017/9781009150200

[7] Devereux S Sabates-Wheeler R. Transformative Social Protection. Brighton: Institute of Development Studies (2004).

[8] Foster G. Under the radar: community safety nets for AIDS-affected households in sub-Saharan Africa. AIDS Care. (2007) 19:54–63. 10.1080/09540120601114469 17364388

[9] Stephen D Duma A. The role of social protection in the socio-economic development of Uganda. J Soc Dev Afr. (1995) 10:5–12.

[10] Davies S. Drought, food insecurity and early warning in Mali. in Adaptable Livelihoods. London: Palgrave Macmillan (1996). p. 79–108. 10.1007/978-1-349-24409-6_520958654

[11] Mkhwanazi N Manderson L. (Eds.). Connected Lives: Families, Households, Health and Care in South Africa. Capetown: HSRC Press (2020).

[12] Makiwane M Gumede NA Makoae M Vawda M. Family in a changing South Africa: structures, functions and the welfare of members. South Afr Rev Sociol. (2017) 48:49–69. 10.1080/21528586.2017.1288166

[13] Heady P Grandits H. “Kinship and social security” in European comparison: rationale and research plan of an EU-funded project. In:Irwin S and Nilsen A, editors. 2018 *Transitions to Adulthood Through Recession: Youth and Inequality in a European Comparative Perspective*. Oxfordshire: Routledge (2018).

[14] Wellman B Wortley S. Different strokes from different folks: community ties and social support. Am J Sociol. (1990) 96:558–88. 10.1086/229572

[15] Durkheim E. (1951). Sociologie et Philosophie. Presses Universitaires de France.

[16] Forster LE Stoller EP. The impact of social support on mortality: a seven-year follow-up of older men and women. J Appl Gerontol. (1992) 11:173–86. 10.1177/073346489201100204

[17] Kawachi I Colditz GA Ascherio A Rimm EB Giovannucci E Stampfer MJ. A prospective study of social networks in relation to total mortality and cardiovascular disease in men in the U.S.A. J Epidemiol Community Health. (1996) 50:245–51. 10.1136/jech.50.3.245 8935453 PMC1060278

[18] Litwin H. Social network type and health status in a national sample of elderly Israelis. Soc Sci Med. (1998) 46:599–609. 10.1016/S0277-9536(97)00207-4 9460839

[19] Sugisawa H Liang J Liu X. Social networks, social support, and mortality among older people in Japan. J Gerontol. (1994) 49:S3–S13. 10.1093/geronj/49.1.S3 8282987

[20] Berkman LF. The role of social relations in health promotion. Psychosom Med. (1995) 57:245–54. 10.1097/00006842-199505000-00006 7652125

[21] Berkman LF Glass T Brissette I Seeman TE. From social integration to health: durkheim in the new millennium. Soc Sci Med. (2000) 51:843–57. 10.1016/S0277-9536(00)00065-4 10972429

[22] Fratiglioni L Wang HX Ericsson K Maytan M Winblad B. Influence of social network on occurrence of dementia: a community-based longitudinal study. Lancet. (2000) 355:1315–9. 10.1016/S0140-6736(00)02113-9 10776744

[23] Seeman TE. Social ties and health: the benefits of social integration. Ann Epidemiol. (1996) 6:442–51. 10.1016/S1047-2797(96)00095-6 8915476

[24] Wellman B. Applying network analysis to the study of support. Soc Netw Soc Support. (1981) 4:171–200.

[25] Barnett GA (Ed.). Encyclopedia of Social Networks. Sage Publications (2011). 10.4135/9781412994170

[26] Jacobson D. The cultural context of social support and support networks. Med Anthropol Q. (1987) 1:42–67. 10.1525/maq.1987.1.1.02a00030

[27] Mann G Delap E. Kinship Care in Sub-Saharan Africa: An Asset Worth Supporting. New York, NY: Bettercare 2000 Network.

[28] Nyambedha EO Aagaard-Hansen J. Changing place, changing position: orphans' movements in a community with high HIV/AIDS prevalence in Western Kenya. In: Children's Places. London: Routledge (2003). p. 166–80.

[29] Kayongo-Male D Onyango P. The Sociology of the African Family. London; New York, NY: Longman (1984).

[30] Nyambedha EO. Change and Continuity in Kin-based Support Systems for Widows and Orphans among the Luo in Western Kenya. Afr Sociol Rev. (2004) 8:139–53. 10.4314/asr.v8i1.23241

[31] Mulvihill K. Why More Women than Men Are Dying in the Ebola Outbreak. (2014). Available online at: https://www.graphic.com.gh/international/international-news/why-more-women-than-men-are-dying-inthe-ebola-outbreak.html (accessed November 23, 2022).

[32] Kenya National Bureau of Statistics. The 2019 Kenya Population and Housing Census: Population by County and Sub-County. Kenya National Bureau of Statistics (2019).

[33] Oxfam. Gendered Patterns of Unpaid Care and Domestic Work in the Urban Informal Settlements of Nairobi, Kenya, Findings from a Household Care Survey 2019(2019). Available online at: https://kenya.oxfam.org/latest/policy-paper/gendered-patterns-unpaid-care-and-domestic-work-urban-informal-settlements (accessed November 24, 2022).

[34] Umbima KJ. Regulating foster care services: the Kenyan situation. Child Welfare. (1991) 70:169–74. 2036871

[35] Drah B. Orphans in sub-Saharan Africa: the crisis, the interventions, and the anthropologist. Africa Today. (2012) 59:2–21. 10.2979/africatoday.59.2.3

[36] Freeman M Nkomo N. Guardianship of orphans and vulnerable children. A survey of current and prospective South African caregivers. AIDS Care Psychol Socio Med Aspects AIDS/HIV. (2006) 18:302–10. 10.1080/09540120500359009 16809107

[37] Carsten J (Ed.). Cultures of Relatedness: New Approaches to the Study of Kinship. London: Cambridge University Press (2000).

[38] Nuttall M. Arctic Homeland: Kinship, Community, and Development in Northwest Greenland (No. 2). Canada: The University of Toronto Press (1992).

[39] Schweitzer PP (Ed.). Dividends of Kinship. London: Routledge (2000).

[40] Howell S. The Kinning of Foreigners: Transnational Adoption in a Global Perspective. Oxford: Berghahn Books (2007). 10.2307/j.ctt1x76frr

[41] Morduch J Sharma MP. Strengthening Public Safety Nets: Can the Informal Sector Show the Way? (No. 583-2016-39570). FCND Briefs 122, International Food Policy Research Institute (2001).

